# Platelet clotting defects in severe trauma identified by a whole blood microfluidic assay

**DOI:** 10.1111/trf.70198

**Published:** 2026-04-02

**Authors:** Gian Rivera Crespo, Jack Killinger, Christopher Bresette, Susan M. Shea, David Ku

**Affiliations:** ^1^ George W. Woodruff Department of Mechanical Engineering Georgia Institute of Technology Atlanta Georgia USA; ^2^ Department of Bioengineering University of Pittsburgh Pittsburgh Pennsylvania USA; ^3^ Trauma and Transfusion Medicine Research Center, Department of Surgery University of Pittsburgh Pittsburgh Pennsylvania USA

**Keywords:** microfluidic assay, platelet dysfunction, primary hemostasis, shear‐induced platelet aggregation, trauma hemorrhage

## Abstract

**Background:**

Hemorrhage is the leading cause of preventable death in severe trauma, with primary hemostasis relying on platelet‐rich clot formation under high shear flow. This study quantifies the incidence, severity, and heterogeneity of platelet dysfunction in Level I trauma patients.

**Study Design and Methods:**

A whole blood assay mimicking arterial hemorrhage fluidic conditions was used to measure platelet‐rich clot formation in Level I trauma patients. Blood samples from patients were collected at arrival and tested for defects in platelet clotting immediately and after 12, 24, and 48 h. Additional clinical parameters such as injury severity, complete blood counts, and blood product use were collected.

**Results:**

The high shear assays were completed in under 5 min using 3 mL of blood. All trauma patients exhibited severe defects in platelet clotting at various time points compared to control blood from healthy donors (*p* < .01). Platelet function varied over time, with some patients exhibiting initial hyper‐clotting followed by dysfunction at 24 h, while others showed persistent impairment for the entire 48 h.

**Discussion:**

The rapid assay was able to distinguish heterogeneous platelet dysfunction in Level I trauma patients. The assay enabled real‐time tracking of a patient's platelet clotting function and response to interventions. By identifying patients with impaired hemostatic function, this assay could potentially inform targeted resuscitation strategies to improve trauma care outcomes.

**Abbreviations**
BMI: body mass index
BUN: blood urea nitrogen
CBC: complete blood count
CV: coefficient of variation
EDTA: ethylenediaminetetraacetic acid
HCT: hematocrit
HGB: hemoglobin
HSV: hue saturation value
IQR: interquartile range
IRB: Institutional Review Board
ISS: injury severity score
LTA: light transmission aggregometry
PDMS: polydimethylsiloxane
PFA‐100: platelet function analyzer‐100
PRBCs: packed red blood cells
RBC: red blood cells
ROTEM: rotational thromboelastometry
SIPA: shear‐induced platelet aggregation
TEG: thromboelastography
TIC: trauma induced coagulopathy
T‐TAS: total thrombus formation analysis system
UPMC: University of Pittsburgh Medical Center
VWF: von Willebrand Factor
WBC: white blood cells

## 1 INTRODUCTION

Traumatic injury remains one of the leading causes of mortality both in the United States and worldwide. Each year approximately 3 million people in the United States require emergency medical care for trauma, with around 200,000 dying from injury‐related causes.^1^ Among these, uncontrolled hemorrhage is the leading causes of preventable death, accounting for approximately 40% of trauma‐related deaths worldwide, most of which occur within the first 24 h post‐injury.^2^, ^3^ In cases of severe trauma, the body's hemostatic response—the process that prevents and stops bleeding—can become dysregulated, resulting in either coagulopathy or hypercoagulation, both of which increase mortality risk.^4^, ^5^

A standard test for trauma‐induced coagulopathy (TIC) is currently unavailable.^5^, ^6^ Traumatic injuries often result in high‐flow hemorrhage, which is associated with extremely high (superphysiological) shear rates.^7^ Shear rates around bleeding wounds caused by arterial puncture or transection can exceed 3000 s^−1^, creating flow conditions that are not compatible with coagulation that typically requires stagnant flow or low flow environments as described by Virchow's triad.^8^ In these high‐shear settings, primary hemostasis is characterized by the rapid formation of a platelet plug. We and others have demonstrated that platelets can aggregate to form macroscopic occlusions when high shear rates are present, in a process known as shear‐induced platelet aggregation (SIPA).^9^, ^10^ The ability of platelets to participate in SIPA is heavily influenced by the functionality of Von Willebrand Factor (VWF) and specific platelet receptors, such as the glycoprotein Ib receptor.^11^, ^12^

**FIGURE 1:**
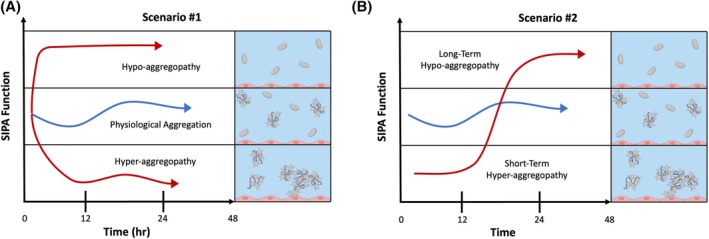
Hypothetical platelet function responses to trauma. (A) Severe trauma may create a hypo‐aggregopathy by a functional defect in shear‐induced platelet aggregation (SIPA). (B) Alternatively, trauma may cause a short‐term hyper‐aggregopathy followed by longer term hypo‐aggregopathy. A rapid, clinical assay of SIPA function should identify which of these scenarios is present in patients with severe trauma.

The lack of a diagnostic platform that can rapidly evaluate SIPA under physiological conditions is a significant gap in trauma care. Formation of strong clots through platelet‐VWF interactions at high shear is essential for effective hemostasis during hemorrhage.^13^ Therefore, a diagnostic test for hemostasis should assess both general impairment of platelet accumulation and the ability to form strong, macroscopic clots capable of arresting bleeding under physiological flow conditions. Additionally, trauma patients display considerable heterogeneity in their hemostatic responses, with some exhibiting hyper‐aggregation and others hypo‐aggregation as illustrated in Figure 1.^14^, ^15^, ^16^ These variations can evolve over time and are further influenced by interventions such as transfusions, anesthesia, and systemic treatments.^17^ A rapid and inexpensive point‐of‐care assay to determine hemostatic function may facilitate the identification and monitoring of TIC evolution in trauma patients.^16^

The primary objective of this work is to evaluate whether a microfluidic SIPA diagnostic test, designed to measure high‐shear platelet aggregation, can detect clotting dysfunction in patients experiencing severe trauma in a clinical setting. The specific goals were to compare the performance of the test in measuring SIPA functionality in healthy individuals and level 1 trauma patients; assess multiple potential endpoints measured by the test to determine their relevance and feasibility for clinical decision‐making; and ensure that the test provides rapid (<10 min) results that can be easily interpreted and utilized by surgeons and clinicians in trauma settings. We hypothesized that some patients with severe trauma would exhibit significant defects in platelet function, as assessed by a microfluidics test of SIPA, and that this dysfunction could be quantified by measuring the formation of occlusive platelet‐rich clots within 10 min.

## 2 STUDY DESIGN AND METHODS

### 2.1 Experimental design

A microfluidic device was designed to recreate the high shear environment associated with hemorrhagic injury. The test section contains two critical requirements: high shear rate and collagen exposure. Blood from healthy subjects and trauma patients was perfused through the test section with the formation of hemostatic occlusion measured by clotting time, end volume, and clot visualization by microscopy.

### 2.2 Microfluidic construction

The microfluidic test section was constructed using polydimethylsiloxane (PDMS) (Dow, Sylgard 184), a material selected for its blood biocompatibility, optical clarity, and ease of fabrication. PDMS molds were cast from a master template to ensure uniformity in the geometry of the test section. Details of the microfluidic geometry were previously described.^18^ Briefly, the microfluidic has four rectangular channels 475 μm wide, 250 μm tall that can be run in parallel with a common inlet. These channels have a stenotic region where the height of the channel ramps down to a stenosis with a height of 70 μm and a length of 800 μm. The stenosis mimics a hole in an artery that experiences high shear bleeding. After curing at a temperature of 50°C for 8 h, the PDMS molds were carefully removed, cleaned, and inspected for geometric consistency. Following inspection, inlets and outlets were added to the microfluidic chips and the PDMS layer was irreversibly bonded to a glass slide using oxygen plasma treatment. The surface of the test section was coated with a solution of fibrillar collagen (Chrono‐log) and allowed to incubate for >2 h at room temperature.

A constant pressure differential across the microfluidic test section was achieved by creating a fluid height difference of 22 cm (16 mmHg pressure difference), maintained by an inlet reservoir containing up to 6 mL of blood to create an initial shear rate of 5000/s. This reservoir setup enabled consistent flow conditions throughout the test duration.

### 2.3 Data collection

The outlets of the microfluidic device were connected to individual outlet reservoirs equipped with precision mass balances. Changes in blood mass over time were recorded and converted into flow rate and volume measurements. These data served as the basis for calculating two key endpoints: clotting time and end volume. Clotting time was defined as the first time where the maximum mass value was achieved, and end volume was defined as the maximum mass value.

Simultaneously, platelet accumulation within the stenotic region was monitored using a dissecting microscope coupled with a high‐resolution camera. Video data captured during the test was processed using an image analysis pipeline involving edge detection and an Hue Saturation Value color gradient analysis. The pipeline distinguished platelet aggregates from the background and calculates the percentage of the surface area covered by aggregates (the surface coverage endpoint). Briefly, images were converted to an HSV color space and thresholded to create a binary image of the low saturation regions (platelet aggregates). Edge detection was applied to the binary image to outline aggregate boundaries. Percent surface coverage was calculated by dividing the area of the binary platelet aggregates by the total stenosis area.

### 2.4 Blood sample collection

This study was conducted under the protocol approved by the University of Pittsburgh Institutional Review Board (IRB#21110126). Pages to the trauma team with basic information regarding incoming patients and activation level were screened by the study team. Four trauma patients were recruited upon their arrival at the University of Pittsburgh Medical Center (UPMC) Presbyterian Hospital Emergency Department. Exclusion criteria included those below the age of 18, pregnant women, and those with a history of drug‐related hospitalization. Baseline samples were taken with the initial IV placement at intake when all other lab draws were performed; no patients received any blood products prior to hospital arrival. Patients presenting as Level 1 trauma activations were defined by the UPMC staff by the presence of penetrating trauma, severe hemorrhage, unstable vital signs, neurological dysfunction, among other possible causes. Normal blood samples were taken at the Georgia Institute of Technology from three healthy adult volunteers under IRB Protocol H22428. Exclusion criteria for the healthy controls included those with transmittable blood diseases, suspected anemia, pregnancy, or those who had taken aspirin or any antiplatelet medication within 10 days of the blood draw.

**FIGURE 2:**
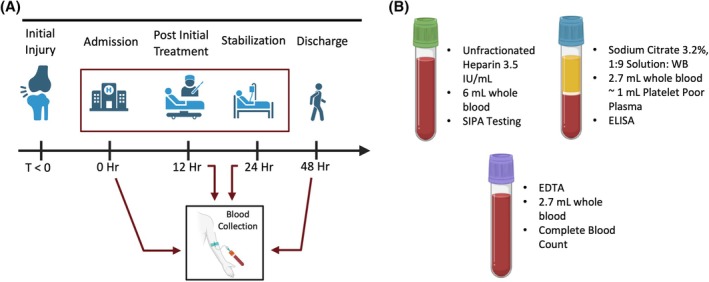
Study design. (A) Patients were enrolled within 4 h of injury, with the first blood sample collected upon hospital arrival (0 h). Follow‐up samples were taken at 12, 24, and 48 h intervals where feasible, to capture dynamic changes in shear‐induced platelet aggregation (SIPA) function. All samples were processed in the microfluidic system within 1 h of collection to minimize variability due to time‐dependent degradation. (B) Blood samples for microfluidic testing were collected in 6 mL vacutainers containing 3.5 IU/mL of low molecular weight heparin for SIPA testing. Additional samples were collected in Vacutainers containing sodium citrate (2.7 mL) for coagulation and platelet testing and ethylenediaminetetraacetic acid (EDTA) (2.7 mL) for complete blood count analysis.

For trauma patients, three tubes were collected: a 6 mL vacutainer containing 3.5 IU/mL of low molecular weight heparin for SIPA testing; a 2.7 mL ethylenediaminetetraacetic acid (EDTA) vacutainer for complete blood count (CBC); and a 2.7 mL sodium citrate vacutainer for platelet count and function testing. A baseline (0 h) sample was collected immediately upon arrival. Patient characteristics upon arrival are summarized in Table 1 and comparisons between clinical data, CBC data, and assay endpoints are summarized in Tables S1 and S2. Follow‐up blood draws were scheduled at 12 ± 2, 24 ± 2, and 48 ± 2 h post‐arrival. The study workflow is summarized in Figure 2. All phlebotomists were given the same instructions and blood samples designated for testing were processed within 1 h of collection to ensure sample integrity and minimize time‐related noise. Each blood sample was tested on the same equipment by one operator, using a single device with four independent microchannels providing four replicates for statistical rigor.

**TABLE 1:** Patient characteristics at arrival.

	Patient 1	Patient 2	Patient 3	Patient 4
Age	54	46	66	86
Sex (year)	Male	Male	Male	Male
Body mass index	25.1	21.3	26.7	26.3
Systolic blood pressure on arrival (mmHg)	160	120	166	140
Heart rate on arrival (bpm)	100	74	77	88
Temperature on arrival (°C)	36.6	36.1	37.3	35.9
Glasgow Coma Scale Score	14	15	15	15
Injury Severity Score	5	5	17	4
Blood urea nitrogen, 0 h (mg/dL)	16	16	22	13
Platelet count, 0 h (10^3^/μL)	268	235	201	248
White blood cells count, 0 h (10^3^/μL)	8.18	17.77	12.12	15.03
Red Blood Cells count, 0 h (10^6^/μL)	6.10	4.55	5.12	4.08
Hemoglobin, 0 h (g/dL)	15.8	13.3	14.8	14.2
Hematocrit, 0 h (%)	51.6	39.9	44.0	39.9

### 2.5 Statistical methods

All statistical analyses were conducted using the PyStats library. Endpoint data were analyzed using the Mann–Whitney–Wilcoxon two‐sided test, with Benjamini–Hochberg corrections used where applicable to account for multiple comparisons. Statistical significance was defined as a corrected *p*‐value of <.05. Results were reported as medians with interquartile ranges for non‐parametric data and as means ± standard deviations for parametric data.

## 3 RESULTS

### 3.1 Clotting time is impaired in trauma patients

**FIGURE 3:**
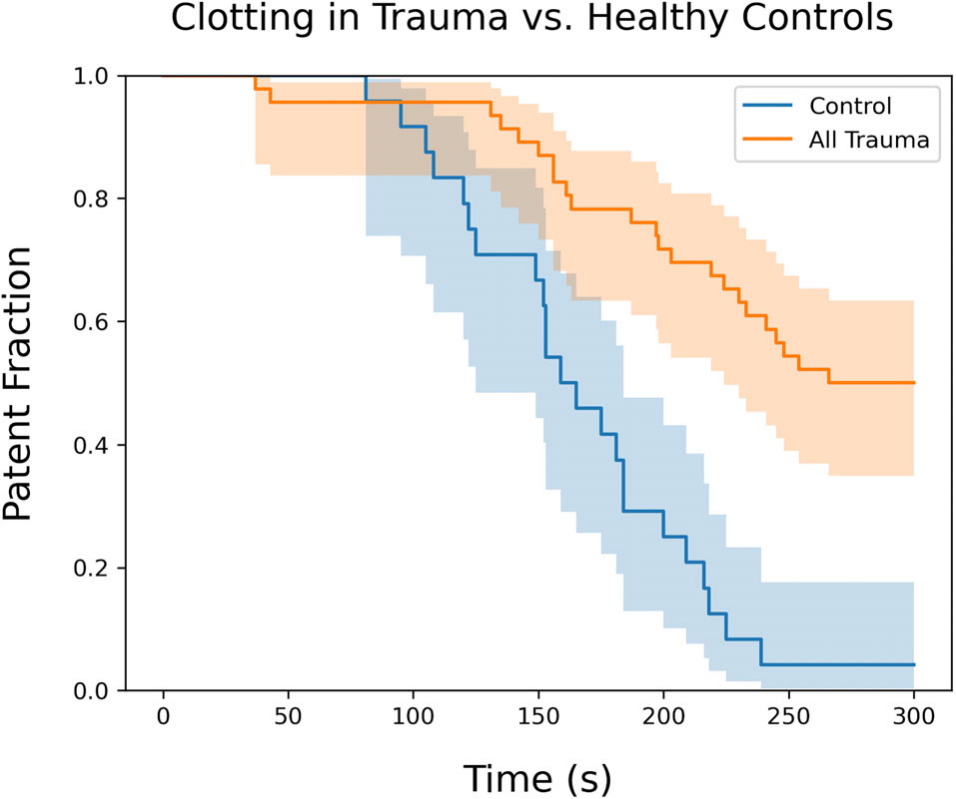
Clotting time survival curves clotting times for aggregated Level 1 trauma patients (orange, *n* = 45 samples) are significantly elongated compared to healthy controls (blue, *n* = 24 samples) as measured using the microfluidic device (*p* < .05). Survival is defined by the presence of measurable blood flow through the test channel. For trauma patients, results from all time points (initial and follow‐ups) are aggregated. Twenty one of forty five of trauma samples did not achieve hemostatic occlusion within 300 s, compared to 1/24 of the healthy controls. Taken together, these changes indicate a delayed or impaired hemostatic response with severe trauma. Shaded regions represent 95% confidence interval.

The microfluidics system generates hemostatic, platelet‐rich clots under conditions generating high shear rates of greater than 5000 s^−1^.^19^, ^20^ The flow rates are consistent with those seen in serious arterial or venous wounds.^8^ A survival plot comparing occlusion times for normal controls and patients with severe trauma is shown in Figure 3. The healthy control group demonstrates a rapid decline in the fraction of patent channels, indicating effective clot formation with an average clotting time of 167 ± 52 s (*n* = 24, from three distinct donors). The steep slope of the control curve, with all but one sample clotting prior to 250 s, reflects rapid hemostasis under normal physiological conditions and demonstrates a functioning hemostatic response in healthy controls using the microfluidic system.

In contrast, blood from the trauma group displays a delayed and incomplete clotting response (Figure 3). For trauma patients, results from all time points (initial and follow‐ups) are aggregated (*n* = 45, from four distinct patients). When the test terminates at 300 s, 46.6% of the trauma group blood samples had not clotted and showed minimal signs of surface aggregation, demonstrating impaired platelet aggregation or clot formation under high‐shear conditions. The two survival curves are statistically significant (Kaplan–Meier estimator, *p* < .05), highlighting the hemostatic dysfunction in trauma patients. Average clotting times for the trauma patient samples was 242 ± 72 compared to 167 ± 52 s in the normal controls (*p* < .001).

### 3.2 End volume provides an alternate endpoint for measuring platelet function

**FIGURE 4:**
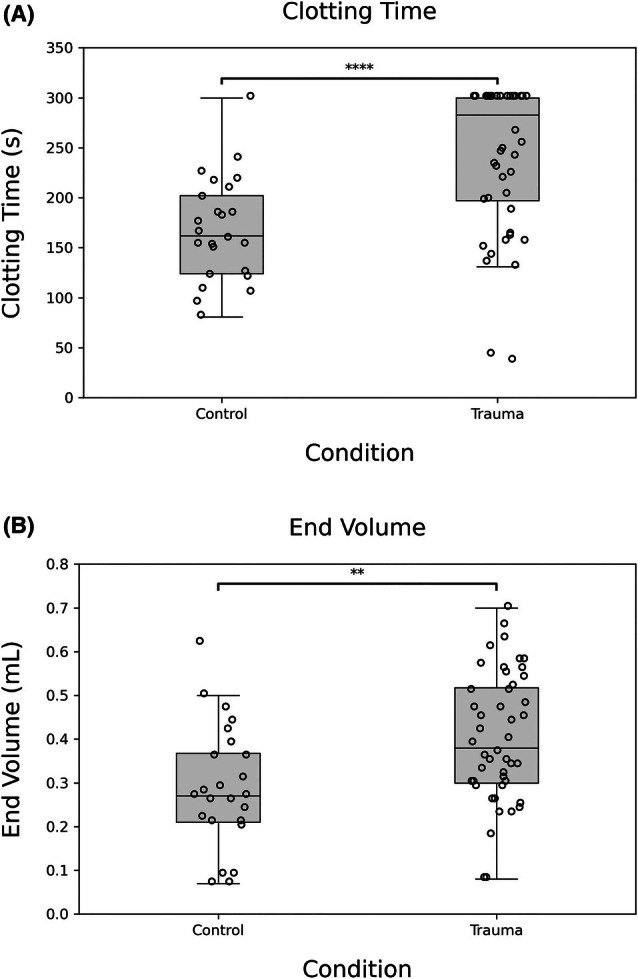
Trauma patients have higher clotting times and end volumes than healthy controls. (A) Clotting times are elongated for trauma patients compared to healthy controls under high‐shear flow conditions. Normal controls exhibit an average clotting time of 167 ± 52 s, with minimal non‐clotting cases, indicative of an efficient platelet aggregation response. Trauma patients demonstrate an average clotting time of 242 ± 72 s (****p < .001), highlighting substantial dysfunction in shear‐induced platelet aggregation and impaired hemostatic function. (B) Demonstrates the end volume endpoint, which measures the total blood volume passing through the microchannel during testing. Healthy controls achieve an average end volume of 0.29 ± 0.14 mL, reflecting effective microchannel occlusion due to aggregation. Trauma patients show a significantly elevated average end volume of 0.40 ± 0.14 mL (**p < .01), with impaired occlusive platelet aggregation.

In addition to clotting time, end volume, or the volume of blood passing through the assay prior to occlusion was measured. The end volume was 400 ± 140 μL for the trauma blood versus 290 ± 140 μL for normal controls (Figure 4, *p* < .01). The end volume was less discriminatory than occlusion time in detecting platelet dysfunction, as evidenced by the greater overlap between the trauma and control populations. However, end volume affords a simpler, more direct readout for the clinician that can be easily read even before the test has completed.

### 3.3 Platelet aggregation changes over time in trauma patients

**FIGURE 5:**
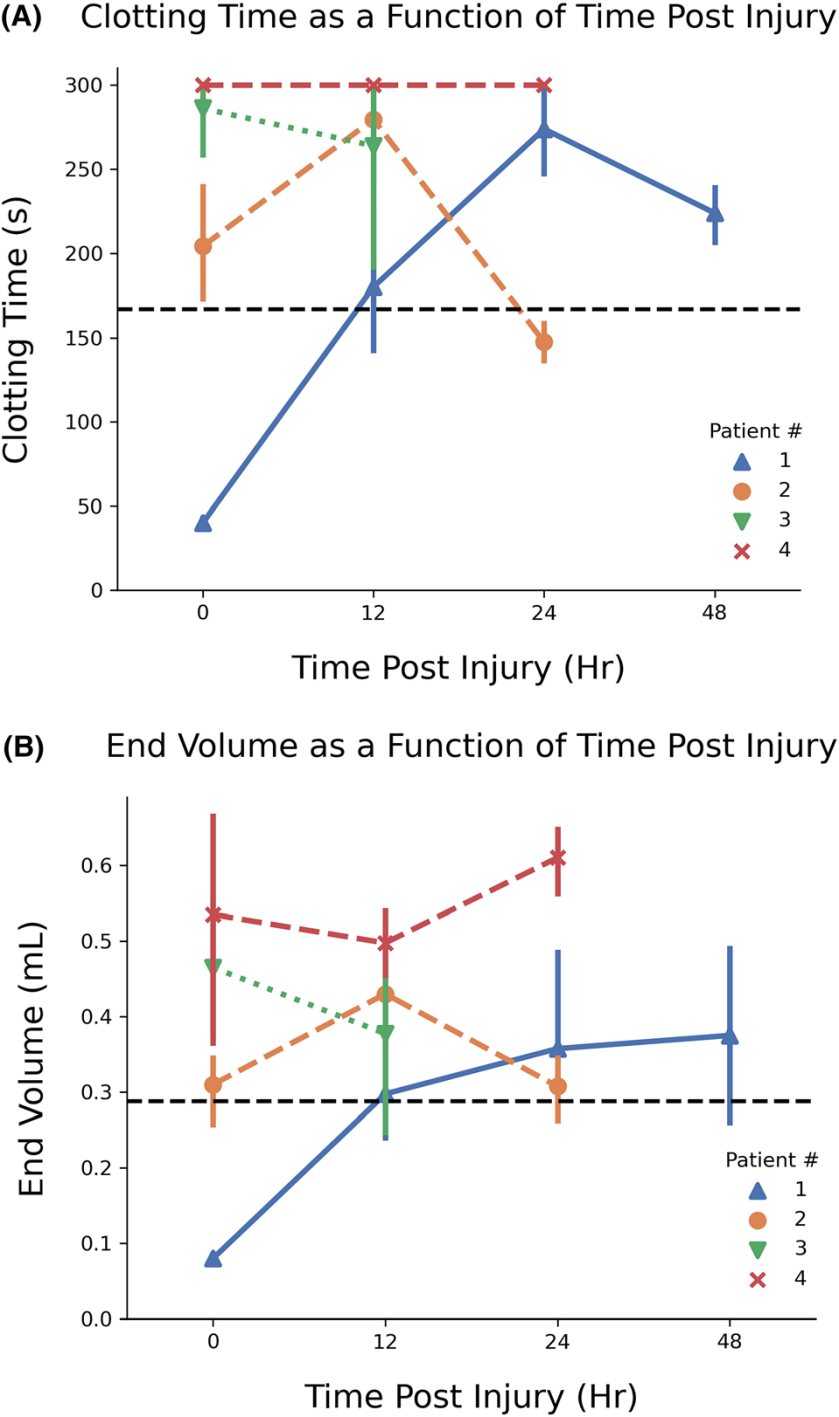
Evolution of clotting time and end volume in individual patients. (A) Clotting time and (B) end volume measurements highlight significant variability in shear‐induced platelet aggregation (SIPA) dysfunction across trauma patients, tracked from intake to 48 h post‐injury. Patient 1 presented in a hyper‐aggregative state at intake, with short clotting times and efficient occlusion, but exhibited a marked decline in SIPA function at 24 and 48 h, indicated by prolonged clotting times and increased end volumes consistent with delayed platelet dysfunction. Patient 2 displayed moderate SIPA dysfunction upon arrival, with elevated clotting times and end volumes that worsened at 12 h but showed recovery by 24 h, reflecting a transient impairment possibly linked to reversible trauma‐induced factors. Patients 3 and 4 demonstrated severe and sustained SIPA dysfunction, with clotting times consistently reaching the 300‐s test maximum and significantly elevated end volumes at all time points, indicative of a profound and persistent loss of hemostatic function. Black dashed lines represent median clotting times and end volumes for the healthy controls and the gray boxes represent the Interquartile Range.

Platelet dysfunction varied over time within individual patients. Figure 5 shows the evolution in clotting time and end volume measurements for different trauma patients post‐injury. Trends in platelet dysfunction were not consistent between patients. Patient 1 had hyper‐aggregopathy on admission with a clotting time of 45 s and end volume of <0.1 mL that is significantly faster than the normal controls (*p* < .05). However, after 24 h, this patient showed little clotting with a clotting time near the maximum time of 300 s and end volume of 340 μL. In contrast, Patient 2 went from a prolonged clotting time of 280 s at 12 h to a normal clotting time of 148 s at 24 h. Blood from Patient 4 never fully occluded, yielding a maximum clotting time of 300 s and end volumes greater than 0.5 mL for all time points. This temporal variability within trauma patients explains the overlap in control and trauma clotting times seen in the survival curves for Figure 3. Only one patient received blood products between assays, with Patient 2 receiving one unit of Packed Red Blood Cells between the baseline and 12 h assays.

### 3.4 Surface coverage by microscopy is not a good endpoint for occlusive clot formation

The choice of endpoint is critical for SIPA assay design. Each endpoint offers distinct advantages and challenges in terms of measurement complexity, relevance to platelet function, and technical implementation (Table 2).

**TABLE 2:** Comparative analysis of relevance, implementation, and cost of each endpoint used for the shear‐induced platelet aggregation assay device.

Endpoint	Hemostatic relevance	Difficulty of implementation	Cost
Clotting time	High	Moderate	Moderate
End volume	High	Low	Low
Surface coverage	Low	High	High

**FIGURE 6:**
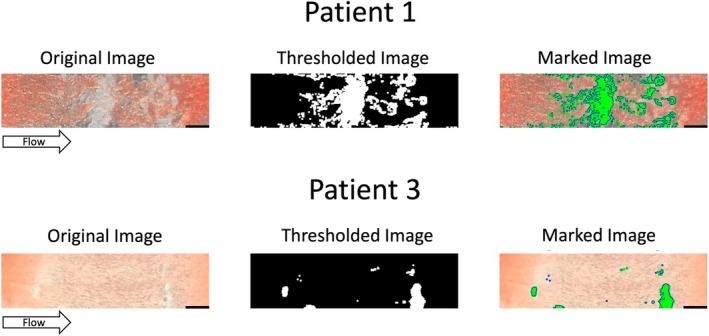
Platelet surface coverage representative images of final platelet aggregation and surface coverage from trauma patients at intake, analyzed under a dissecting microscope (magnification >5×, scale bar 200 μm). Patient 1 exhibited significant surface coverage (25.5%), with platelet aggregates forming dispersed or structured patterns that achieve partial hemostatic occlusion. In contrast, Patients 3 showed minimal surface coverage (4.1%), reflecting severe shear‐induced platelet aggregation (SIPA) dysfunction. Variations in coverage patterns, including localized strips or dispersed platelet aggregates, highlight patient‐specific differences in SIPA function.

Platelet surface coverage in microfluidic test section is widely used to detect platelet adhesion and aggregation in laboratory research and was an alternative endpoint considered. Representative images of platelet surface coverage are shown in Figure 6. Compared to clotting time and end volume, the images were more difficult to collect and analyze automatically. Images clearly show platelet accumulation, but most images fell into one of two categories—either minimal surface coverage or widespread surface coverage. However, within the samples with widespread coverage, it was difficult to distinguish growth on the surface from occlusive growth spanning the height of the test section, even after thresholding. Thus, this endpoint is the most difficult to implement and has accuracy and precision issues when detecting cessation of flow. Microscope images confirmed the presence of platelet‐rich clots, but were not a good continuous variable endpoint to track over time.

End volume has the advantage of being easily measured without the need for electronic sensors. End volume has direct clinical relevance, analogous to bleeding volume during hemorrhage. End volume is more sensitive to samples with mild changes in platelet function because it may distinguish between a sample that begins to form a clot but fails to be occlusive in 300 s and a sample that does not form a clot.

Clotting time is also clinically relevant, with parallels to the amount of time required to achieve hemostasis. However, the endpoint of clotting time requires additional electronics and sensors to automate. Further, the definition of occlusion can vary (90% reduction in flow, no measurable increase in mass, etc.) which has a large impact on the calculation of clotting time.

The coefficient‐of‐variation (CV) for control clotting time measurements in this study was 28%, with an average clotting time of 167 s, illustrating the degree of population variability for this assay. Intra‐subject variability, as measured by CV within individual donors, ranged from 14% to 28%, demonstrating good repeatability. For end volume, the CV averaged 47% for controls, with a per donor CV ranging from 26% to 71%. Notably, the differences observed between control patients and trauma patients exceeded the assay variability, indicating that the disparities reflect true physiological differences rather than measurement noise. Thus, the SIPA assay can reliably distinguish between control and trauma patients with impaired platelet aggregation.

The correlation between clotting time and end volume further emphasizes the utility of these endpoints in assessing hemostatic function (*r* ^2^ = 0.58). While clotting time offers more precise discrimination for average and near‐average cases, end volume can distinguish between moderate and severe non‐clotting events without requiring additional blood.

## 4 DISCUSSION

A diagnostic platform capable of assessing whole‐blood SIPA functionality under high shear conditions would address several key challenges in trauma care:

1. Rapid assessment of hemostatic function: Identifying patients at risk of hemorrhagic complications or thrombotic events based on their SIPA functionality.
2. Monitoring temporal changes: understanding how SIPA evolves during the acute phase of injury and in response to treatments.
3. Personalized treatment planning: Tailoring hemostatic interventions based on individual SIPA functionality, optimizing the use of blood products and hemostatic agents.

This preliminary study demonstrates that traumatic injury is associated with measurable changes in high‐shear platelet aggregation function, which may impair hemostasis and contribute to hemorrhage. Platelet dysfunction was detected and quantified using an in vitro microfluidics assay that mimics primary hemostasis via Shear Induced Platelet Aggregation (SIPA). Among Level 1 trauma patients, half exhibited prolonged clotting times and increased end volumes, indicated impaired platelet aggregation. Notably, this defect could develop over 24 h in patients who had normal clotting upon admission, while others showed a persistent dysfunction over 48 h. Decreased platelet surface coverage observed by brightfield microscopy further confirmed the loss of platelet aggregation function.

Rapid measurement of primary hemostatic function could assist in the triage and treatment of patients with severe trauma. Some patients with bleeding but preserved hemostatic function may spontaneously stop bleeding and only require observation. Resources can be focused instead on patients with a severe bleeding defect that may require being taken to the operating room for surgical closure. Patients with clotting defects may be candidates for limited hemostatic blood products such as platelets and cryoprecipitate. This assay for platelet clotting would be a simple addition to the diagnostic workup of the patient with severe trauma and visible or suspected hemorrhage.

The results presented in this study underscore the role of platelet function for primary hemostasis in Level 1 trauma patients. Several assays exist to assess platelet function, including Light Transmission Aggregometry (LTA),^21^ Verify now, PFA‐100, and T‐TAS. However, these are not typically used to evaluate TIC. Viscoelastic assays, such as Rotational Thromboelastometry (ROTEM) and thromboelastography (TEG), may be used to predict trauma outcomes but do not directly measure platelet function.^22^, ^23^, ^24^, ^25^ Furthermore, all these tests require significant pre‐conditioning of blood samples, such as anticoagulation with citrate or the addition of chemical agonists, which can artificially alter platelet function and interfere with the assessment of SIPA.^26^, ^27^ Catastrophic loss of hemostatic SIPA function has only recently been investigated in patients suffering from severe traumatic injury.^15^, ^28^, ^29^, ^30^ Using the assay described here, SIPA can now be studied and tracked following admission to the trauma bay, setting the stage for additional preclinical research on the role of SIPA over time in trauma. This study provides the first evidence that SIPA function in trauma patients varies significantly over time and between different patients, ranging from hyper‐ to hypo‐aggregatory dysfunction within the same patient. All patients tested had suffered severe Level 1 blunt or penetrating trauma. While the study did not focus on the predictive power of the assay, we hypothesize that hemostatic complications will be more likely to occur in patients with severe dysfunction in future studies. We also hypothesize that patient and clinical factors such as treatment with blood products, medication affecting platelet activation, surgery, or inflammation from infection would alter clotting times measured by our assay and require distinct studies.

The microfluidic assay allowed for rapid testing of all samples within 10 min, which is critical for timely decision‐making after trauma admission. Clinicians may benefit from objective data on hemostatic function to guide urgent decisions, such as the need for transfusion products or surgical intervention. While current treatment regiments rely on TEG/ROTEM results to interpret one aspect of platelet function, these assays fail to recreate the high‐shear condition of SIPA and can take up to 60 min to get results. In contrast, the presented microfluidic platform provides faster results that may more accurately predict SIPA dysfunction.

Multiple end points were compared with the assay, each with differing levels of complexity and sensitivity. While previous studies on platelet aggregation function have primarily focused on microscopy and fluorescent tagging of platelets and VWF to visualize and quantify aggregation, this approach is complicated for a clinical assay. Microscopy provides valuable insight into the occurrence of aggregation and spatial characteristics of the resulting clots, but does not measure hemostatic occlusion. With standard microscopy it is difficult to account for clot volume (height in the *z*‐direction) and to estimate flow. Most published work using microscopic imaging to quantify clotting function focuses on surface level aggregation.^31^ However, an occlusive clot requires significant platelet‐VWF volume growth and sufficient mechanical integrity to counteract arterial pressures.^13^, ^31^

The results refine our understanding of trauma‐induced “coagulopathy” by emphasizing the importance of platelet hemostasis, which is independent of coagulation defects. Our assay specifically detects defects in platelet function, enabling early identification of hemostatic impairments, that could impact morbidity and mortality in trauma patients. Future research should focus on address the current endpoint limitations and validate these assays in larger, more diverse trauma populations. Investigating the underlying mechanisms driving SIPA variability and dysfunction in trauma patients may also inform targeted therapies. While other microfluidic systems (T‐TAS, Stasys) are being studied in trauma, our system's ability to form large, occlusive clots may better represent global platelet function.^31^, ^32^, ^33^ Specifically, the size of these assays means that platelet‐surface interactions play a larger role than platelet‐platelet interactions, and the constant flow used to generate high shear introduces noise through embolization.^34^, ^35^ By overcoming these challenges, microfluidic assays have the potential to transform the management of trauma‐induced SIPA dysfunction and improve patient outcomes through timely, precise interventions.

The current study has several limitations. The low number of patients should be followed with a larger series of patients. Nonetheless, the results from this pilot demonstrate the high sensitivity of the test to identify changes in clotting dysfunction over time. Additionally, while trauma is a highly heterogeneous population, all four of the injured patients included in this study were male and three of the four had low Injury Severity Scorescores of 5 or 4. Future work is necessary to expand these findings to a more diverse and severely injured population. Two of the three control subjects were also male, representing 20/24 of the samples tested, with ages ranging from 28 to 35. A control group that skews male and younger mirrors the general trauma population; however, because of low N, the trauma patients recruited were significantly older.^36^ The quality of the assay is directly affected by the quality of the blood samples and blood collection can present logistical challenges that introduce variability. While controls were not on antiplatelet drugs due to exclusion criteria, trauma patient antiplatelet status at intake was unknown and not collected during the study. For controls, blood was drawn using a syringe into 3.5 IU/mL of heparin to minimize anticoagulant effects, while trauma patient's samples were collected in custom vacutainers filled with 3.5 IU/mL heparin. Vacutainers were sometimes underfilled by up to 50%, effectively doubling the heparin concentration in those samples. However, previous work showed no significant difference in occlusion times between samples with 3.5 versus 8 IU/mL heparin, and with all samples a sufficient quantity of blood was collected for running a valid assay.^37^ Vacutainers with 15 IU/mL of heparin are commonly available but may alter clotting times and are similarly susceptible to underfilling. Citrate vacutainers, commonly used for coagulation and CBCs, have been shown to produce abnormal platelet function and may not be suitable for SIPA testing.^38^, ^39^ Another limitation is that the assay was not compared to clinical metrics such as estimated blood loss, pre‐treatment hemoglobin levels, or blood product use. While the assay is expected to correlate with these values, further clinical studies are needed to confirm these associations in a more homogenous population, such as penetrating trauma with and without massive transfusion protocol activation.

## 5 CONCLUSIONS

A rapid, primary hemostasis assay was developed to mimic the hemodynamics of hemorrhage. This clinical diagnostic test detected defects in platelet clotting in patients who had severe, Level 1 trauma relative to blood from healthy donors (*p* < .001). Clotting in these patients was variable at admission and evolved over the 48 h of observation with results ranging from hyper‐clotting to no measurable clotting. This SIPA‐based microfluidics assay may have clinical value in differentiating patients with defects in platelet primary hemostasis and directing targeted care.

## CONFLICT OF INTEREST STATEMENT

GRC, CAB, and DNK are listed as inventors on a patent publication related to the technology described in the manuscript, PCT/US2024027245.

## Supporting information


**Table S1.** Baseline demographic, injury, transfusion, and clinical outcome characteristics for the study trauma patients.

**Table S2.** Longitudinal complete blood count and related hematology measurements for the study trauma patients.

## Data Availability

The data that support the findings of this study are available from the corresponding author upon reasonable request.
