# Supplementary material for: Platelet clotting defects in severe trauma identified by a whole blood microfluidic assay
Source: Transfusion. 2026 Apr 2;66(Suppl 1):S205–15. doi: 10.1111/trf.70198 (PMC13184437; doi:10.1111/trf.70198)
Supplement: Supplementary file 1 — Table S1. Baseline demographic, injury, transfusion, and clinical outcome characteristics for the study trauma patients. [file TRF-66-S205-s002.pdf]

Study patient ID	042	043	044	045
Date and time of arrival	9/6/24 18:32	9/7/24 14:12	9/7/24 15:50	9/7/24 18:14
Age	54	46	66	86
Sex	Male	Male	Male	Male
Race	Black	White	White	White
BMI	25.1	21.3	26.7	26.3
Systolic blood pressure on arrival	160	120	166	140
Heart rate on arrival	100	74	77	88
Temperature on arrival	36.6	36.1	37.3	35.9
Glasgow Coma Scale score	14	15	15	15
Mechanism of injury (blunt/penetrating)	MVA rollover	GSW to suprapubic region	Helmeted MCA on raceway	Fell ~ 15-20 feet down a hillside; +etoh
Injury severity score	5	5	17	4
Blood products given?	None	Yes	None	None
Blood product type and amount		PRBC, 1 unit		
Blood product date time		9/7/24 14:41		
ICU length of stay	0	0	1	1
Hospital length of stay	4 days	18 days	2 days	2 days
Mortality (yes/no)	No	No	No	No
Bleeding complications	None	None	None	None
Thrombotic complications	None	None	None	None
BUN, 0hr	16	16	22	13
BUN, 12hr	11	13	16	10
BUN, 24hr	8	12	16	12
pH, 0hr	7.48		7.35	
